# Effect of breathing exercises on oxidative stress biomarkers in humans: A systematic review and meta-analysis

**DOI:** 10.3389/fmed.2023.1121036

**Published:** 2023-04-05

**Authors:** Ting-ting Li, Hong-ying Wang, Hui Zhang, Ping-ping Zhang, Ming-chen Zhang, Hai-yang Feng, Xiao-yong Duan, Wen-bo Liu, Xiao-wen Wang, Zhong-guang Sun

**Affiliations:** ^1^School of Rehabilitation Medicine, Weifang Medical University, Weifang, China; ^2^Weifang People’s Hospital and Brain Hospital, Weifang, China; ^3^School of First Clinical Medical, Weifang Medical University, Weifang, China; ^4^Weifang People’s Hospital, Weifang, China; ^5^Affiliated Hospital of Weifang Medical University, Weifang, China

**Keywords:** breathing exercises, oxidative stress, malondialdehyde, superoxide dismutase, glutathione, nitric oxide

## Abstract

**Background:**

Breathing exercises improve oxidative stress in healthy young adults and patients with diabetes, hypertension, and chronic obstructive pulmonary disease. Furthermore, the mechanism of respiratory intervention is controversial. Therefore, in this meta-analysis, we aimed to systematically evaluate the effects of breathing exercises on oxidative stress biomarkers in humans and provide evidence for the clinical application of breathing exercises.

**Methods:**

The Embase, PubMed, Cochrane Library, Web of Science, CNKI, and WANFANG databases were searched for studies about the effects of breathing exercises on human oxidative stress levels, with no restraints regarding time, race, or language. The experimental group included various breathing exercises, and the outcome index included malondialdehyde, superoxide dismutase, and glutathione, nitric oxide, vitamin C, or total antioxidant capacity levels from a randomized controlled trial. Data were extracted by more than two authors and reviewed by one author.

**Results:**

Ten studies were included from five countries. Data from patients with no disease, chronic obstructive pulmonary disease, hypertension, or diabetes were included. Participants who performed breathing exercises had greater changes in the included biomarkers than those who did not, suggesting that these biomarkers can be used to evaluate oxidative stress after respiratory interventions.

**Conclusion:**

Breathing exercises increased SOD and GSH activities and decreased MDA content.

**Systematic review registration:**

https://www.crd.york.ac.uk/prospero/display_record.php?ID=CRD42022337119, identifier CRD42022337119.

## 1. Introduction

Oxidative stress (OS) is the imbalance between the production of the reactive oxygen species (ROS) by oxidation and the clearance of antioxidants in organisms (1, 2), which leads to the accumulation of free radicals, vascular endothelial lipid peroxidation, and inflammatory reactions in the body (3–6). ROS consist of O_2_ and oxygen-containing molecules that have not been completely reduced (7). ROS are mainly produced in mitochondria and released into the mitochondrial matrix, intermembrane space, and cytoplasm (8–10). A small amount of ROS promotes cell proliferation, although a large quantities accumulated ROS negatively affects cell functions, resulting in lipid peroxidation that produces malondialdehyde (MDA) and, ultimately, cell death (4). Superoxide dismutase (SOD), which is widely distributed in the body, plays an important role in protecting the body from injury by dismutating the superoxide ions in internal and external environments and is an important free radical scavenger in the body (11). Reducing glutathione (GSH) combines with superoxide and free radicals to resist ROS damage to thiol groups, protecting the cell membrane that contains sulfhydryl proteins and sulfhydryl enzymes and preventing free radical damage to important organs (12). Nitric oxide (NO) bioavailability–which is mediated by ROS *via* endothelial NO synthase (eNOS) activation–leads to chronic inflammatory responses and cardiovascular injury (13).

When the body is exposed to harmful stimuli, the oxidant-antioxidant imbalance leads to the imbalance of musculin breakdown and synthesis, ultimately resulting in varying degrees of skeletal muscle atrophy and thereby leading to decreased muscle strength, which affects exercise endurance and quality of life (14). *Via* the attenuation of the mitochondrial oxidative respiratory chain and cell death, inflammatory cells activate and accumulate a large number of free oxygen radicals to promote the lipid generation of MDA, aggravating the damage of OS (15–17). Changes in the OS index are related to harmful stimulation, aging, and disease progression (18–20). The diagnosis of a disease or chronic inflammation is associated with changes in OS markers and, often, with an increase in antioxidant biomarker levels and a decrease in oxidative stress biomarker levels as the disease ameliorates (21–28). OS is measured by analyzing the composition of urine, sputum, blood, and exhaled gas (29–31). The lipid peroxidation product MDA, antioxidant enzyme SOD, and non-enzyme GSH are the most commonly used indexes (2, 32).

Moderate-intensity training improves OS while high-intensity training does not reverse OS and muscle damage during aerobic exercises (33–37). Breathing exercises, a moderate-intensity training technique used in pulmonary rehabilitation, have been widely used in clinical practice (38). By prolonging the breathing time, slowing the breathing rate, and reducing the alveolar-arterial oxygen gradient, breathing exercises enhance the strength and endurance of the respiratory muscles to increase pulmonary ventilation and improve the efficiency of gas exchange (39–44). These exercises effectively relieve the body of hypoxia and improve endurance, maintaining the overall health of the body.

Breathing exercises improve OS in healthy young adults and patients with diabetes, hypertension, and COPD (12, 45–53). However, the sample sizes of the previous studies were small, and the impact of respiratory interventions on patients without primary lung diseases is also controversial (54, 55). Therefore, in this meta-analysis, we aimed to systematically evaluate the effects of breathing exercises on OS biomarkers in humans to provide evidence for the clinical application of breathing exercises.

## 2. Methods

The meta-analysis was registered with PROSPERO (registration number: CRD42022337119).

### 2.1. Data sources and eligibility criteria

The Embase, PubMed, Cochrane Library, Web of Science, CNKI, and WANFANG databases were searched for randomized controlled trials regarding the effects of breathing exercises. There were no restraints in terms of time, race, or language. The keywords “breathing exercise” and “oxidative stress” were used to link the corresponding free words for the advanced retrieval of studies. In two Chinese databases, Chinese vocabulary retrieval was used to obtain Chinese studies (Table 1).

**TABLE 1:** Electronic database search strategies.

Electronic database	Search stragety	
	Mesh	Text word
Embase/pubmed/ cochrane/web of science	Breathing exercises	Exercise, breathing; respiratory muscle training; muscle training, respiratory; training, respiratory muscle
	Oxidative stress	Oxidative stresses; stress, oxidative; antioxidative stress; antioxidative stresses; stress, antioxidative; anti-oxidative stress; anti oxidative stress; anti-oxidative stresses; stress, anti-oxidative; oxidative damage; damage, oxidative; oxidative damages; oxidative stress injury; injury, oxidative stress; oxidative stress injuries; stress injury, oxidative; oxidative injury; injury, oxidative; oxidative injuries; oxidative cleavage; cleavage, oxidative; oxidative cleavages; oxidative dna damage; dna damage, oxidative; damage, oxidative dna; oxidative dna damages; dna oxidative damage; dna oxidative damages; damage, dna oxidative; oxidative damage, dna; oxidative and nitrosative stress; oxidative nitrative stress; nitrative stress, oxidative; oxidative nitrative stresses; stress, oxidative nitrative; nitro-oxidative stress; nitro oxidative stress; nitro-oxidative stresses; stress, nitro-oxidative; stresses, and nitro-oxidative
CNKI	[Subject: breathing exercise (accurate)] OR [Subject: breathing rehabilitation (accurate)] OR [Subject: inspiratory muscle training (accurate)] AND [Subject: oxidative stress (accurate)] OR [Subject: oxidation-antioxidation (accurate)] OR [Subject: oxidation reduction state (accurate)]	
WANFANG	[Subject: (breathing exercise) or Subject: (breathing rehabilitation) or Subject: (inspiratory muscle training)] and [Subject: (oxidative stress) or Subject: (oxidation-antioxidation) or Subject: (oxidation reduction state)]	

### 2.2. Inclusion criteria

Randomized controlled trials in which the intervention was breathing training, such as slow and fast deep breathing, a simple prototype respiratory muscle trainer, diaphragmatic breathing exercises, pranayama of yoga, straight leg raising breathing, inspiratory muscle training, or time-efficient inspiratory muscle strength training and the outcome indexes of OS were MDA, SOD, GSH, NO, vitamin C, or total antioxidant capacity (TAC) levels were included in this meta-analysis. The participants of the included studies were healthy individuals or patients with COPD, hypertension, or diabetes.

Studies that were not randomized controlled trials, including abstracts of conference papers, and those with missing data or data that could not be extracted or obtained from the author were excluded from the meta-analysis. Studies that used non-blood outcome indicators such as urine, sputum, and exhaled gas and those that did not include OS outcome indexes were also excluded. If the clinical randomized controlled trial design was determined as not rigorous, or the Jadad score was <3, the study was not included in the meta-analysis. Moreover, studies in which the breathing exercises were not included in the experimental group were excluded from the meta-analysis.

### 2.3. Study selection and data collection processes

Data extraction, including the title, author, year, country, the population in each group, intervention methods, intervention time, and outcome index data, was performed by two authors. Mean ± standard deviation values were directly extracted, while the data included in the figures were extracted using error bar graphs with error lines. Disputes were resolved by the examiner, and a consensus was achieved through discussion.

### 2.4. Quality evaluation

Quality assessment was independently performed by two authors using the Cochrane risk-of-bias tool to assess the literature grade based on the low risk criteria for A, B, and C. In cases of disagreement, the authors reevaluated their judgment and reached an agreement through discussion.

### 2.5. Statistical analysis

The extracted data were meta-analyzed using RevMan 5.4 and Stata 16.0 software. Continuous variables are presented as weighted mean differences (MD) or e mean differences, and the 95% CI was determined. Bias risk maps and forest maps were created. When *I*^2^ was 50% or less, the fixed-effects model was used. When *I*^2^ was more than 50%, the heterogeneity was considered to be large, and a sensitivity analysis was performed to reduce the heterogeneity. Statistical significance was set at *P* < 0.05.

## 3. Results

### 3.1. Description of studies

**FIGURE 1:**
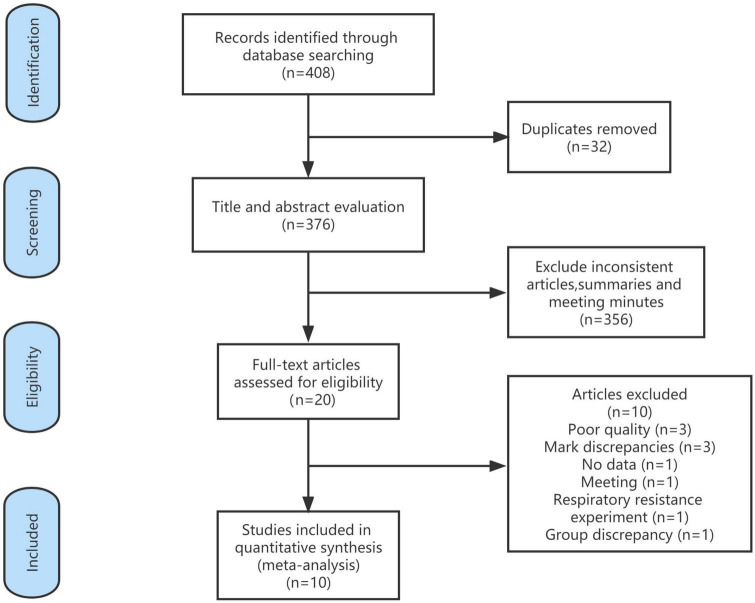
Flow diagram.

A total of 408 articles were identified in the database search. Of these, 32 duplicate articles were excluded and 356 were excluded based on the title and abstract. A total of 20 articles were read in full, and 10 articles were included in this meta-analysis. The references of the included studies were searched for additional studies, but none met the inclusion criteria (Figure 1).

The 10 studies included in this meta-analysis were conducted in five different countries and included 519 participants, including 267 in the treatment group and 252 in the control group (Table 2). Breathing exercises included slow and fast deep breathing (52), a simple prototype respiratory muscle trainer (51), diaphragmatic breathing exercises (47), pranayama of yoga (45, 46, 48, 49), straight leg raising breathing (50), inspiratory muscle training (13), and time-efficient inspiratory muscle strength training (53). The shortest intervention time was 20 days (53), and the longest was 3 months (46, 47, 50). The earliest study was published in 2002 and revealed the changes in MDA and SOD levels in healthy, young individuals after pranayama yoga breathing interventions (45). Two studies were conducted in Thailand (51, 52), one in South Korea (48), five in India (45–47, 49, 50), one in the United States (14), and one in China (53). The most recent studies (13, 53) included inspiratory muscle training exercises. Patients with COPD (51–53), diabetes (46, 47, 49), and hypertension (13, 50) were included in some studies, and two studies included healthy, young adults (45, 48). The main indexes were MDA (45–48, 50–53), SOD (45–50, 53), GSH (46, 47, 49–53), and NO levels (13, 48, 51, 52). TAC (51, 52) and catalase (CAT) (48), vitamin C (46, 47, 50), and F2-isoprostane levels (48) were also measured in some studies (Table 2).

**TABLE 2:** Characteristics of included studies.

References	Country	Population and number (treatment/control group)	Intervention of treatment group	Intervention time	Oxidative stress biomarkers
Leelarungrayub et al. (52)	Thailand	Chronic obstructive pulmonary disease 30 (15/15)	Fast deep breathing techniques	1 month; twice daily, morning and evening	MDA, GSH, NO, TAC
Leelarungrayub et al. (51)	Thailand	Chronic obstructive pulmonary disease 20 (10/10)	A simple prototype respiratory muscle trainer	6 weeks; 1–5 daily sessions of 15–30 min	MDA, GSH, NO, TAC
Lim and Cheong (48)	Korea	Young healthy People 25 (12/13)	voluntary regulation of breath (pranayama)	12 weeks; 1 day a week for 90 min	MDA, SOD, GSH, NO, CAT
Pati et al. (50)	India	Grade-1 Hypertension 57 (28/29)	Breathing practices, Straight leg raising breathing, Pranayama	3 months; 6 days in a week for 1 h	MDA, SOD, GSH, Vitamin C
Hegde et al. (47)	India	Type 2 diabetes 123 (60/63)	Diaphragmatic breathing exercise	3 months; twice daily for 15–20 min	MDA, SOD, GSH, Vitamin C
Mahapure et al. (49)	India	Diabetics 40 (30/10)	Pranayama (breathing exercises)	6 weeks; 6 days in a week for 1 h	SOD
Bhattacharya et al. (45)	India	Young healthy males 60 (30/30)	Pranayama (breathing exercises)	10 weeks; daily for 30 min	MDA, SOD
Hegde et al. (46)	India	Type 2 diabetes 40 (20/20)	Pranayama (breathing exercises)	3 months; 6 days in a week for 75–90 min	MDA, SOD, GSH, Vitamin C
Craighead et al. (13)	USA	Midlife/Older adults with above-normal blood pressure 36 (18/18)	Time-efficient inspiratory muscle strength training	6 weeks; 6 days per week	NO, TAS
Xu et al. (53)	China	Acute exacerbation of chronic obstructive pulmonary disease 88 (44/44)	Inspiratory muscle training	20 days; twice daily for 30 min	MDA, SOD, GSH

MDA, malonaldehyde; SOD, superoxide dismutase; GSH, glutathione; NO, nitric oxide; TAC, total antioxidant capacity; CAT, catalase; TAS, total antioxidant status.

### 3.2. Risk of bias in included studies

**FIGURE 2:**
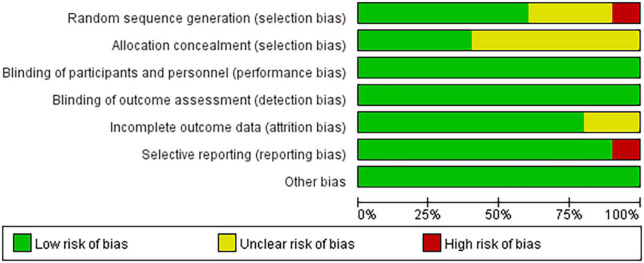
Risk of bias graph.

**FIGURE 3:**
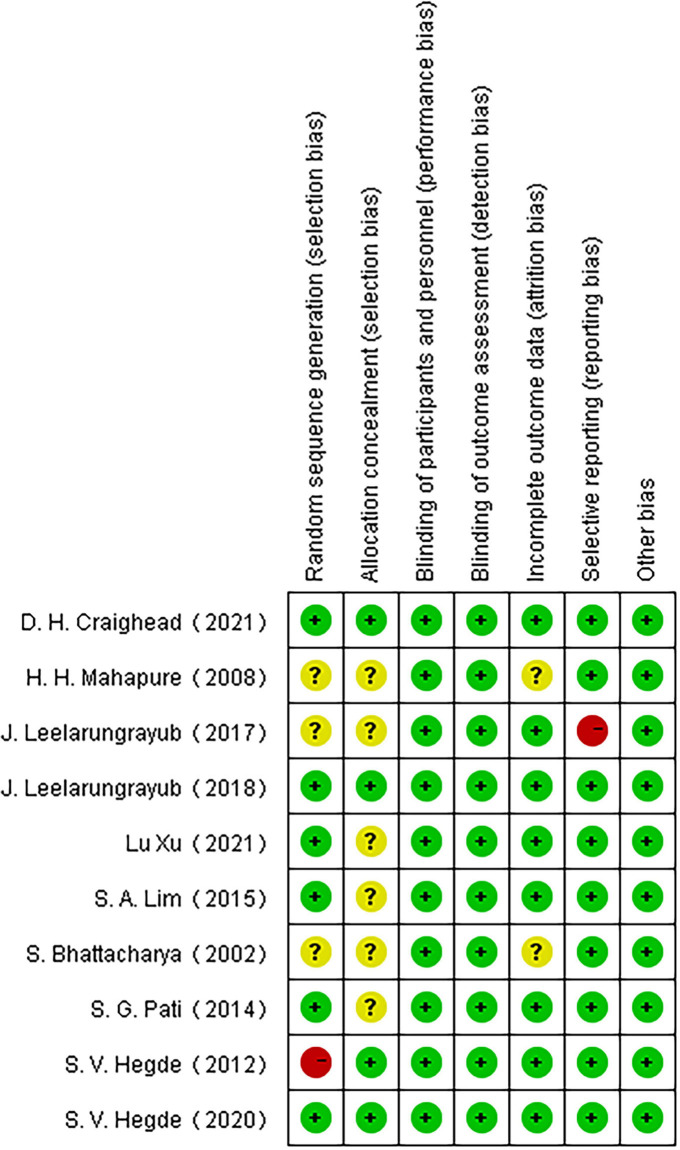
Risk of bias summary.

Among the 10 included articles, three were grade A and seven were grade B. Studies that did not explicitly refer to randomized trials and allocation concealment were included in the unclear category. One study (47) did not use randomness and was considered to have a high risk of bias for the generation of random sequences. Another study (51) regarding the application of a simple prototype respiratory muscle trainer for breathing exercises did not include a complete explanation of the experimental design (Figures 2, 3).

### 3.3. Meta-analysis

#### 3.3.1. MDA

**FIGURE 4:**
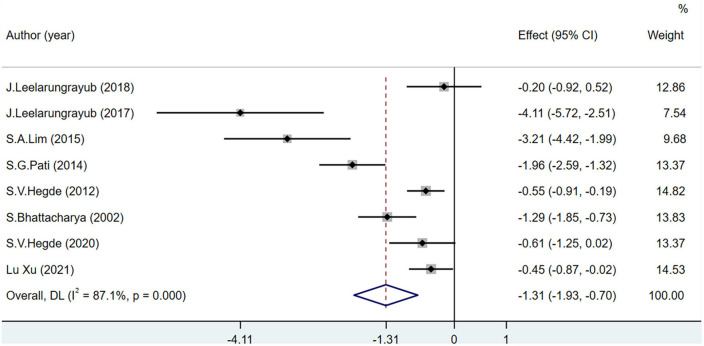
Effects of breathing exercises on malondialdehyde levels.

The MDA level was used as the main OS index in eight studies including 443 participants. MDA levels decreased significantly more in the experimental group than in the control group (MD = −1.31; 95% CI = −1.93 to −0.7; *P* < 0.001) (Figure 4).

#### 3.3.2. SOD

**FIGURE 5:**
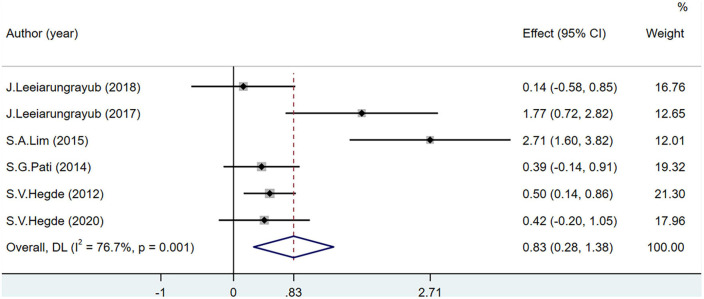
Effects of breathing exercises on superoxide dismutase levels.

The SOD level was used as an indicator of OS in seven studies including 433 participants. *I*^2^ was greater than 50%. The increase in SOD levels was significantly greater in the experimental group than in the control group (MD = 1.55; 95% CI = 0.53–2.57; *P* < 0.001). However, after removing one study (48) from the sensitivity analysis, there was no significant difference in the levels of SOD between the groups (MD = 0.73; 95% CI = −0.03–1.50; *P* = 0.001) (Figure 5).

#### 3.3.3. GSH

**FIGURE 6:**
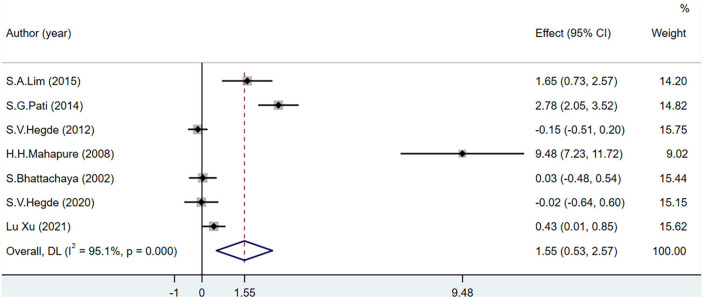
Effects of breathing exercises on glutathione levels.

Glutathione levels were recorded in seven studies including 383 participants. One study (53) may have included bias as the heterogeneity reduced after its removal (*I*^2^ = 76.7%). The GSH level of the experimental group was significantly higher than that of the control group (MD = 0.83; 95% CI = 0.28–1.38; *P* < 0.001) (Figure 6).

#### 3.3.4. NO

**FIGURE 7:**
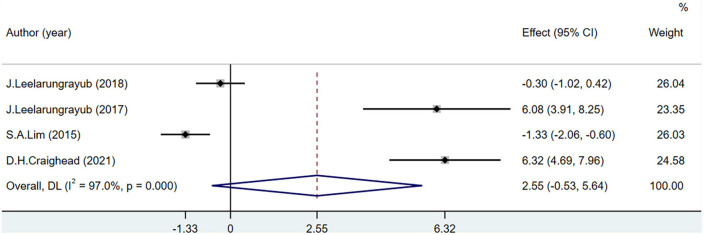
Effects of breathing exercises on nitric oxide levels.

Four studies including 111 participants reported NO level. The NO levels were not significantly different between the groups (MD = 2.55; 95% CI = –0.53 to 5.64; *P* < 0.001) (Figure 7).

#### 3.3.5. Vitamin C

**FIGURE 8:**
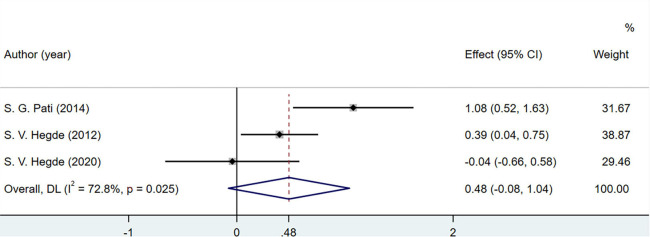
Effects of breathing exercises on vitamin C levels.

A total of three studies involving 220 patients discussed the changes in vitamin C levels in the human body. Vitamin C concentration did not change significantly (MD = 0.48; 95% CI = −0.08 to 1.04; *P* = 0.025) (Figure 8).

#### 3.3.6. TAC

**FIGURE 9:**
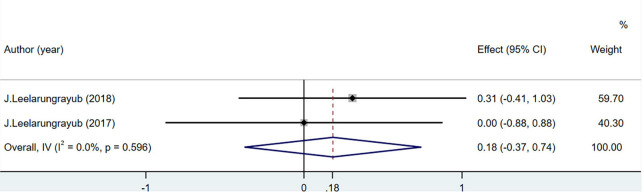
Effects of breathing exercises on total antioxidant capacity levels.

Total antioxidant capacity levels were measured in 50 people in two studies. The difference was not statistically significant (MD = 0.18; 95% CI = –0.37 to 0.74; *P* = 0.596) (Figure 9).

## 4. Discussion

Breathing exercises have been a method of rehabilitation of patients with respiratory system diseases for decades (56). Respiratory muscle training has been proven to improve lung function in healthy participants and in patients with COPD, hypertension, diabetes, and chronic kidney disease (57), as it increases exercise endurance and the overall quality of life. OS, as an indicator to measure the survival and mortality of human aging and later disease stages (26, 58–60), is a hotspot of current research, although few studies regarding the impact of breathing exercises on OS have been conducted. The role of breathing exercises in aging or disease progression can be investigated as the effect of respiratory interventions on OS indexes.

In this study, MDA, SOD, and GSH levels, the main outcome indexes of breathing exercises for OS, were investigated. Breathing exercise increased SOD and GSH activities and decreased MDA content. SOD is converted into hydrogen peroxide by catalyzing oxygen reduction, which is then converted to water by GSH. It can effectively remove oxygen free radicals, protect endothelial cells from damage, relax vascular smooth muscle by increasing NO release, thus achieving the purpose of lowering OS (48). Participants who performed breathing exercises had greater changes in these biomarkers than those who did not, suggesting that these biomarkers can be used to evaluate OS after respiratory interventions.

Malondialdehyde is an oxidation product. MDA levels decreased significantly more in participants who performed breathing exercises than in those who did not (45, 47, 48, 50, 51, 53). This may be due to the fact that MDA is the end product of lipid peroxidation (32), a reaction caused by oxygen free radicals by attacking polyunsaturated fatty acids of biofilms. MDA participates in various physiological and pathological processes in the body (4). Leelarungrayub et al. (52) and Hegde et al. (46) reported a significant decrease in MDA levels in the experimental and control groups in their studies but no statistical differences between the groups. In two studies (35, 54), non-aerobic exercises as part of the slow deep breathing method and yoga required a certain intensity of training, and both improved the body’s antioxidant capacity and reduced the MDA content compared with those in the control groups (35), accounting for the lack of significant differences between the groups. These results are similar to those of another study (54) that compared breathing exercises to aerobic exercises in patients undergoing hemodialysis.

In two studies (45, 47), SOD levels decreased in patients with diabetes who performed breathing exercises. This decrease may be due to the adverse effects of long-term, low-intensity training on the body’s antioxidant capacity (61). Chronically increased blood glucose levels increase the content of glycation end products in the body, leading to increased oxidase activity, self-oxidation of glucose, and glycosylation of proteins, resulting in OS reactions (62–64). However, Mahapure et al. (49) reported that SOD levels increased significantly in patients with diabetes performing breathing exercises, which may be related to yoga exercises that are more intense than the breathing exercises. High-intensity exercises resulted in increased SOD levels in patients with diabetes, improving the OS state (65). After a sensitivity analysis and the exclusion of this previous study, the total effect size was not statistically significant in this meta-analysis. In healthy participants and patients with hypertension and COPD (45, 48, 50, 53), SOD dismutates the superoxide anions to generate hydrogen peroxide and oxygen during stable breathing exercises, protecting the body from damage caused by the internal and external environments and maintaining normal physiological activities (11).

The total effect size of the increase in GSH levels was significant in participants performing breathing exercises in this meta-analysis; however, Leelarungrayub et al. (51, 52) concluded that there was no significant change in GSH levels in patients with COPD. This may be due to the training method or the disease period of the population of the included studies. Another study (53) used a Drager Evi-ta-II ventilator to conduct respiratory interventions on patients with acute exacerbations of COPD and reported increased GSH levels, which improved the antioxidant capacity of the body. However, the sensitivity analysis in this study showed that there may be some bias in the previous study. In studies regarding the increase in GSH levels in patients with metabolic diseases and in healthy participants (46–48, 50), GSH improved the antioxidant defense mechanism by reducing the hydrogen peroxide dismutated from SOD into water during breathing exercises (12).

In this analysis, the variation of NO levels was the biggest difference, which may be due to the specific exercise programs in each included study. Moderate exercise increases the secretion of NO by endothelial cells, while long-term and high-intensity exercise can reduce the secretion of NO by endothelial cells (65). In patients with COPD, different respiratory interventions have resulted in conflicting data (13, 48, 51). These differences may also be related to the stimulation degree of endothelial cells in different disease states (66). A high NO level in patients with COPD is related to the severity of the disease and airflow obstruction (67). Breathing exercises stimulate endothelial cells to reduce NO secretion, which helps to prevent deterioration due to the disease (52). In patients with hypertension, oxidation imbalance leads to the production of a large quantity of ROS (68). The bioavailability of NO is increased by the activation of eNOS with ROS, improving OS (13). Under normal conditions, no changes in OS will occur in healthy individuals, and the increase in NO levels will cause damage to the tissues and cells of the body (69). Breathing exercises do not increase NO levels in healthy individuals (48). Therefore, when NO is used as an indicator of OS in different physiological states of the body, its influence on overall OS should be further determined according to the reaction mechanism in the body.

Total antioxidant capacity and CAT, vitamin C, and F2-isoprostane levels were not included in the meta-analysis. TAC and CAT levels were not significantly different between the groups in three studies (48, 51, 52), which may be due to the low sensitivity of these indicators or because respiratory interventions did not result in significant changes. Vitamin C may not be an accurate biomarker of oxidation-antioxidant status, as it can be obtained through food and vitamins (70, 71). Of the 10 studies included in this meta-analysis, one (48) showed the serum F2-isoprostane levels in healthy individuals and reported that the experimental group had lower levels than the control group. The F2-isoprostane content in exhaled air among patients with COPD increased after breathing exercises were performed but decreased after 6 min of walking (72). This may be due to the high variability and small sample size in the previous study. Rapid or gentle breathing regulates the contraction and relaxation of skeletal muscle, which can affect the F2-isoprostane content in exhaled air (73, 74). Aerobic exercise has been reported to reduce F2-isoprostane levels in patients with circulatory system or metabolic diseases (75, 76). However, the effects and action mechanism of breathing exercises on F2-isoprostane remain unknown. Future studies evaluating the role of F2-isoprostane in the OS state are needed to confirm the reference values in patients performing breathing exercises.

In the early stages of aging and diseases, the body reacts to harmful environmental stimuli by increasing the antioxidant levels (77). However, when these levels exceed the antioxidant capacity of the body, exogenous ROS cause inflammation (78), stimulate the release of endogenous superoxide and hydrogen peroxide to produce OS, and lead to a series of pathological reactions such as mitochondrial disorders, lipid peroxidation, and apoptosis. Inflammatory and pathological reactions further promote OS, creating a vicious cycle that worsens the disease (79). Breathing exercises are steady and medium-intensity exercises (54, 74), which effectively inhibit lipid peroxidation reactions, maintain the antioxidant capacity at a high level, enhance the stability of the internal environment, and promote the removal of harmful substances (80, 81).

### 4.1. Study limitations

Owing to the limited number of published articles, the specific methods of respiratory interventions and disease types were not strictly distinguished in this study. The differences in conclusions regarding changes in OS indicators in the included studies may be related to different breathing exercises and disease states included in the studies.

## 5. Conclusion

Breathing exercises can improve the main biological indicators of OS toward the direction of antioxidation and improve the OS state by increasing the levels of antioxidants and reducing those of oxidative markers. In addition, the MDA level is the most commonly used and most sensitive indicator to evaluate the impact of breathing exercises on the OS state. However, the effect of SOD on patients with diabetes and the role of GSH in patients with COPD require further research. The reaction mechanism of NO in patients with different diseases should be considered when it is used as an indicator of OS.

## Data availability statement

The original contributions presented in this study are included in the article/supplementary material, further inquiries can be directed to the corresponding authors.

## Author contributions

TTL: conceptualization, methodology, formal analysis, investigation, and writing—original draft. HYW and HZ: investigation and editing. PPZ: conceptualization and writing—review and editing. MCZ and HYF: investigation. XYD and WBL: supervision. XWW and ZGS: writing—review and editing. All authors contributed to the article and approved the submitted version.
